# HELQ deficiency impairs the induction of primordial germ cell‐like cells

**DOI:** 10.1002/2211-5463.13810

**Published:** 2024-05-08

**Authors:** Cong Wan, Yaping Huang, Xingguo Xue, Gang Chang, Mei Wang, Xiao‐Yang Zhao, Fang Luo, Zhi‐Zhong Tang

**Affiliations:** ^1^ Maoming People's Hospital China; ^2^ State Key Laboratory of Organ Failure Research, Department of Developmental Biology, School of Basic Medical Sciences Southern Medical University Guangzhou China; ^3^ Department of Biochemistry and Molecular Biology Shenzhen University Health Science Center China; ^4^ Guangdong Key Laboratory of Construction and Detection in Tissue Engineering Southern Medical University Guangzhou China; ^5^ Guangzhou Regenerative Medicine and Health Guangdong Laboratory (GRMH‐GDL) China

**Keywords:** germ cells, helicase POLQ‐like, *in vitro* induction, primordial germ cell‐like cells, stem cells

## Abstract

Helicase POLQ‐like (HELQ) is a DNA helicase essential for the maintenance of genome stability. A recent study identified two HELQ missense mutations in some cases of infertile men. However, the functions of HELQ in the process of germline specification are not well known and whether its function is conserved between mouse and human remains unclear. Here, we revealed that *Helq* knockout (*Helq* ^−/−^) could significantly reduce the efficiency of mouse primordial germ cell‐like cell (PGCLC) induction. In addition, *Helq* ^−/−^ embryonic bodies exhibited a severe apoptotic phenotype on day 6 of mouse PGCLC induction. p53 inhibitor treatment could partially rescue the generation of mouse PGCLCs from *Helq* mutant mouse embryonic stem cells. Finally, the genetic ablation of *HELQ* could also significantly impede the induction of human PGCLCs. Collectively, our study sheds light on the involvement of HELQ in the induction of both mouse and human PGCLCs, providing new insights into the mechanisms underlying germline differentiation and the genetic studies of human fertility.

## Graphical abstract

Helicase POLQ‐like (HELQ) is an essential DNA helicase for maintaining genomic stability, and its mutations have been associated with male infertility. We found that absence of HELQ markedly reduces the induction efficiency of mouse primordial germ cell‐like cells *in vitro*. This reduction can be partially reversed by treatment with a p53 inhibitor. Furthermore, HELQ is also implicated in the induction of human primordial germ cell‐like cells.

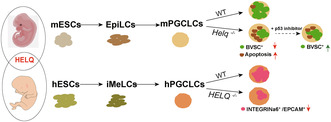

**Abbreviations**
BMP 4: bone morphogenetic protein 4
BV: *Blimp1*‐mVenus
Cas9: CRISPR‐associated protein 9
CRISPR: clustered regularly interspaced short palindromic repeats
Dox: doxycycline
E: embryonic day
EB: embryonic body
EGF: epidermal growth factor
EpiLC: epiblast‐like cell
ESC: embryonic stem cell
FA: Fanconi anemia
FACS: fluorescence‐activated cell sorting
gRNA: guide RNA
hESC: mouse embryonic stem cell
hPGC: human primordial germ cell
hPGCLC: human primordial germ cell‐like cell
ICL: interstrand cross‐link
iMeLC: incipient mesoderm‐like cell
LIF: leukemia inhibitory factor
mESC: mouse embryonic stem cell
mPGC: mouse primordial germ cell
mPGCLC: mouse primordial germ cell‐like cell
PBS: phosphate‐buffered saline
PGCLC: primordial germ cell‐like cell
PGC: primordial germ cell
PSC: pluripotent stem cell
SC: *Stella*‐ECFP
SCF: stem cell factor
ssDNA: single‐stranded DNA
TUNEL: terminal deoxynucleotidyl transferase dUTP nick end labeling
WT: wild‐type

Germline development is a complicated process critical for the continuum of a species. Genetic defects during germ cell development could lead to infertility and increase the susceptibility to germ cell tumors [1]. In mammals, the specification of germline occurs early during embryogenesis, when primordial germ cells (PGCs) arise from the proximal epiblast, this process is in response to bone morphogenetic protein 4 (BMP4) secreted from the extra‐embryonic ectoderm and WNT3 from the visceral endoderm [2, 3]. Subsequently, the specified PGCs migrate and colonize the genital ridge [4, 5]. In mouse, it is well‐established that three transcription factors *Blimp1(Prdm1)*, *Prdm14* and *Tfap2c* form a core regulatory network essential for PGC specification [6, 7]. Human PGCs (hPGCs) retain a core set of genes that are also critical for mouse germ cell development, including specification genes (e.g. *PRDM1*, *PRDM14* and *TFAP2C*) and pluripotency factors (e.g. *NANOG*, *POU5F1* and *DPPA3*) [8, 9], but there are some key differences between mouse and hPGCs. For instance, *SOX17* is critical for hPGCs specification, exerting its function upstream of *BLIMP1*, but is dispensable for that in mouse PGCs (mPGCs) specification [10, 11]. To date, as a result of the technical and ethical limitations with respect to obtaining the rare PGCs from early embryos, especially for hPGCs, the molecular mechanisms underlying these processes have not been fully explored.

Recently, the emergence and rapid development of *in vitro* gametogenesis has made it easier for basic research and offer the potential clinical applications [6, 7, 12, 13, 14]. Remarkably, the induction of cells resembling PGCs has been achieved *in vitro* from pluripotent stem cells (PSCs) [6, 15, 16, 17, 18]. Mouse PSCs were first converted into the epiblast‐like cells (EpiLCs), a cellular state similar to pregastrulating epiblasts. Then, the EpiLCs could further be differentiated into mouse primordial germ cell‐like cells (mPGCLCs) in response to cytokines [including BMPs, leukemia inhibitory factor (LIF), epidermal growth factor (EGF) and stem cell factor (SCF)] [19]. Alternatively, without cytokines, simultaneous overexpression of *Blimp1*, *Prdm14* and *Tfap2c* could also transform EpiLCs into mPGCLCs efficiently [20]. Especially, the stem cell‐derived mPGCLCs could be further induced into functional gametes and give birth to healthy offspring [19, 20]. Similarly, human PSCs could be induced into human primordial germ cell‐like cells (hPGCLCs) through the incipient mesoderm‐like cells (iMeLCs) stage [11, 21]. Using the *in vitro* gametogenesis platform, several key questions in germ cell biology have been resolved [22, 23, 24].

HELQ, also known as HEL308, is an ATP‐dependent 3′‐5′ DNA helicase and was first discovered in the human and mouse genomes through its homology to MUS308 in *Drosphila melanogaster* [25]. *HELQ* deficiency results in the hypersensitivity to DNA cross‐linking agents such as cisplatin and mitomycin C [26, 27]. Previous studies revealed the participation of HELQ in DNA interstrand cross‐link (ICL) repair in a manner distinct from the Fanconi anemia (FA) pathway, which was a dominant mechanism for inter‐strand cross‐link repair in vertebrates [28, 29]. In addition, HELQ promotes homologous recombination at damaged replication forks by directly interacting with the RAD51 paralogue complex BCDX2, modulated by RPA and RAD51 [27, 30]. In humans, *HELQ* is expressed in the heart, skeletal muscle, testes and ovaries [31]. A recent study using a set of 183 infertile men diagnosed with Sertoli cell‐only syndrome discovered the mutation of *HELQ* in six male infertile patients, indicating the correlation between *HELQ* mutation and male infertility [32]. Similarly, *Helq* ^−/−^ mice exhibited subfertility, ICL sensitivity and increased predisposition to ovarian and pituitary tumors. The mutant mice also exhibited phenotypes of smaller testes and dysgenesis/atrophy in ovaries [26]. Additionally, a previous study found that *Helq* ^−/−^ male mice showed a decreased number of PGCs beginning at embryonic day (E)12.5 [33]. However, whether HELQ is involved in specification of PGCs and whether its roles in PGCs development are conserved between mouse and human remain unknown.

## Materials and methods

### Culture of mouse embryonic stem cells (mESCs) and human embryonic stem cells (hESCs)

*Helq* ^−/−^ mESCs carrying an 8‐bp deletion in the both alleles of *Helq* gene were obtained the clustered regularly interspaced short palindromic repeats (CRISPR)/CRISPR‐associated protein 9 (Cas9) technique [33] and maintained as previously described [34]. Briefly, mESCs were cultured in N2B27 medium with 2i‐LIF condition [1 μm PD0325901 (T6189; TargetMol, Boston, MA, USA), 3 μm CHIR99021 (T2310; TargetMol) and 1000 U·mL^−1^ LIF (ESG1107; Merck Millipore, Burlington, MA, USA)] on feeder cells. Cells were passaged every second day. For feeder‐free culture, mESCs were cultured on dishes coated with poly‐l‐ornithine (0.01%; A‐004‐M; Sigma, St Louis, MO, USA) and laminin (300 ng·mL^−1^; 23017015; Invitrogen, Waltham, MA, USA) in N2B27 medium with 0.4 μm PD0325901, 3 μm CHIR99021 and 1000 U·mL^−1^ LIF.

The hESCs were cultured on dishes coated with Matrigel (354277; Corning Inc., Corning, NY, USA) in mTeSR1 (85850; Stemcell, Vancouver, BC, Canada). Cells were passaged every 4–6 days using EDTA and the medium was changed every day.

### Generation of a *HELQ* ^−/−^ cell line

The human *HELQ* ^−/−^ cell line was obtained as described previously [35]. Briefly, to knock out human *HELQ* gene, a pair of oligos encoding guide RNA (gRNA) targeting the site was annealed, phosphorylated, and ligated to the linearized px458 vector [36]. The specific gRNA sequence used to target the human *HELQ* locus was GTGGTTCCCGCATCCGCCGGCGG. Subsequently, 8 μg of pX458 constructs containing gRNA was electroporated into Fy‐hES‐3 cells using a Neon™ Transfection System (MPK10096; Thermo Fisher, Waltham, MA USA). Two days later, the EGFP positive cells were sorted by fluorescence‐activated cell sorting (FACS). The sorted cells were manually picked into Matrigel‐coated 96‐well plates at a density of a single cell per well, and cultured in mTeSR1 medium supplemented with 10 μm ROCK inhibitor [Y‐27632 (1254; Tocris, Bristol, UK)]. After 12–15 days of culture, the surviving clones were passaged into 24‐well plates. Genomic DNA was then extracted from these clones for sequencing.

### Correction of the *Helq* mutation

Gene correction was performed by targeted integration using the homologous recombination‐based strategy as previously described [37]. The gRNA was inserted into the px458 vector [36]. We designed a single‐stranded DNA (ssDNA) oligo containing a synonymous substitution to create a *Sma*I enzyme recognition site. The ssDNA oligo was ordered from RuiBiotech (Nanjing, China) and the sequence was: GCCTGCCGAGGACACGGAGGACGAAGCTGCAGCCGGGAGCCGCCGCCGGAAAACCGGGAGCCCGGGGCACGCCCAGGTACAGCCCTGCTGAGTCCCCGCATCCCCGTTGA. Subsequently, 5 μg of pX458 constructs containing gRNA and 0.2 μm ssDNA oligo were electroporated into *Helq* ^−/−^ mESCs using a Neon™ Transfection System (MPK10096; Thermo Fisher). Two days after transfection, the EGFP positive cells were sorted by FACS. Subsequently, single clones were manually picked and transferred into 96‐well plates. Genomic DNA was extracted from these individual clones. Next, the target locus was amplified, and the PCR products were subjected to *Sma*I enzyme digestion. Successful correction was indicated by the presence of corresponding bands resulting from the cleavage of the PCR product. The PCR product was subcloned into the pClone007 Versatile Simple Vector (007VS; Tsingke, Beijing, China) for further sequencing.

### Induction of EpiLCs and mPGCLCs

The induction of mESCs into EpiLCs and mPGCLCs was performed following the previously published experimental protocols with minor modification [20, 34]. Briefly, for inductionof EpiLCs, 1.0 × 10^5^ mESCs were plated in a 12‐well plate coated with 16.7 mg·mL^−1^ human plasma fibronectin (FC010; Merck Millipore) in EpiLCs medium [N2B27 medium supplemented with 20 ng·mL^−1^ activin A (338‐AC; R&D Systems, Minneapolis, MN, USA), 12 ng·mL^−1^ bFGF (133‐FB; R&D Systems)] and 1% knockout serum replacement (10828028; Gibco, Waltham, MA, USA) for 42–48 h and the medium was changed every day. mPGCLCs were induced from 2.0 × 10^3^ EpiLCs per well under a floating condition in a low cell‐binding, U‐bottom, 96‐well plate (7007; Corning Inc.) in modified mPGCLCs medium [N2B27 with 15% KSR, doxycycline (Dox)] for 6 days and the medium was changed every 2 days. A male cell line was used in this study.

### Induction of hPGCLCs

hPGCLCs were induced from hESCs via iMeLCs as described previously [21]. For the induction of iMeLCs, hESCs were dissociated into single cells with TrypLE Select (Life Technologies, Carlsbad, CA, USA) and plated 3.0 × 10^5^ cells in a 12‐well plate coated with human plasma fibronectin in GK15 medium supplemented with 50 ng·mL^−1^ activin A, 3 μm CHIR99021 and 10 μm of Y‐27632 (1254; Tocris). After 40–48 h, iMeLCs were dissociated into single cells with TrypLE Express (12604013; Gibco) and then plated in a low cell‐binding, U‐bottom, 96‐well plate at 3000 cells per well in the GK15 medium (GMEM with 15% KSR, 0.1 mm NEAA, 2 mm l‐glutamine, 1 mm sodium pyruvate and 0.1 mm β‐mercaptoethanol) supplemented with 200 ng·mL^−1^ BMP4 (314‐BP‐010; R&D Systems), 100 ng·mL^−1^ SCF (255‐SC‐010; R&D Systems), 50 ng·mL^−1^ EGF (236‐EG; R&D Systems), 100 ng·mL^−1^ LIF (7734‐LF‐500’ R&D Systems) and 10 μm Y‐27632 to induce into hPGCLCs.

### Western blot

Whole‐cell extracts were lysed and run on an SDS/PAGE gel. Following electrophoresis, the proteins were transferred to nitrocellulose membranes. The membranes were then incubated in 5% milk and treated with specific antibodies. The primary antibodies used in the present study were: rabbit‐anti‐HELQ (19436; Cell Signaling Technology, Danvers, MA, USA) and rabbit‐anti‐β‐ACTIN (20536; Proteintech, Rosemont, IL, USA). The secondary antibodies used in the present study were: anti‐rabbit HRP (zb2301; ZSJB‐Bio, Beijing, China). The blots were developed using an ECL kit (36208ES60; Yeason, Shanghai, China).

### Immunofluorescence

For immunofluorescence, the ESCs or EpiLCs were fixed in 4% paraformaldehyde in phosphate‐buffered saline (PBS) for 20 min at room temperature. After washing three times in PBS, the samples were permeabilized and blocked in blocking solution comprising 2% BSA and 0.3% Triton X‐100 in PBS for 30 min at room temperature. This was followed by incubation with primary antibodies diluted in blocking solution overnight at 4 °C. Subsequently, the samples were washed with PBS three times and incubated with secondary antibodies for 1 h at room temperature. Relevant Alexa Fluor488/594/647‐conjugated secondary antibodies (dilution 1 : 500; Jackson ImmunoResearch, West Grove, PA, USA) were used for labelling. Nuclei were stained with 10 μg·mL^−1^ Hoechst 33432 for 15 min. Images were obtained using a LSM880 confocal microscope (Zeiss, Oberkochen, Germany).

For immunofluorescence of day 4 embryonic bodies (EBs), EBs induced from wild‐type (WT) or *HELQ* ^−/−^ hESCs were fixed in 4% paraformaldehyde in PBS for 1 h, then washed twice in PBS and incubated with 30% sucrose for 1 h at 4 °C. Next, the samples were embedded in OCT embedding matrix and stored at −80 °C. Subsequently, samples were sliced into 8‐μm cryosections using a cryostat (Leica, Heidelberger, Germany). Slides with cryosections were air dried at room temperature for at least 15 min, followed by the immunofluorescence as described above. The primary antibodies were: rabbit anti‐OCT4 (dilution 1 : 200; ab19857; Abcam, Cambridge, UK), rabbit anti‐NANOG (dilution 1 : 400; ab70482; , Abcam), mouse anti‐OCT4 (dilution 1 : 400; sc‐5279; Santa Cruz Biotechnology, Dallas, TX, USA), rabbit anti‐SOX2 (dilution 1 : 400; Ab97959; Abcam) and rabbit anti‐TFAP2C (dilution 1 : 400; sc‐8977; Santa Cruz Biotechnology).

### Flow cytometry analysis

EBs were dissociated into single cells with 0.25% trypsin for 15–20 min at 37 °C. After washing with PBS containing 1% fetal bovine serum, the cells were suspended in FACS buffer (1% fetal bovine serum in PBS) and filtered using a 40‐μm nylon mesh. For hPGCLCs, cells were incubated with APC‐conjugated anti‐ human CD326 (EpCAM) antibody (#324208; Biolegend, San Diego, CA, USA) and BV421‐conugated anti‐human/mouse CD49f (INTEGRINa6) antibody (#313624; Biolegend) for 15 min at 4 °C. Flow cytometry analysis was performed using the FACS Calibur system (Becton Dickinson, Franklin Lakes, NJ, USA).

### Cell apoptosis analysis

Cell apoptosis analysis was performed using an Annexin V‐FITC/7‐AAD Cell Apoptosis Detection kit (P‐CA‐202; Procell, Bethel, CT, USA). In brief, mouse EBs were dissociated into single cells with 0.25% trypsin for 15–20 min at 37 °C. The single cells were then washed twice with pre‐cooled PBS and suspended with 250 μL of binding buffer. Subsequently, 100 μL of the cell suspension was mixed with 5 μL of Annexin V‐FITC and 10 μL of 7‐AAD solution. The mixture was incubated for 15 min at room temperature. The samples were then analyzed using a flow cytometer (CytoFlex; Beckman, Brea, CA, USA) for further examination. Terminal deoxynucleotidyl transferase dUTP nick end labeling (TUNEL) staining was performed using a One‐step TUNEL *In Situ* Apoptosis Kit (E‐CK‐A322; Elabscience, Houston, TX, USA).

### Statistical analysis

Statistical analysis was carried out using prism (GraphPad Software Inc., San Diego, CA, USA). The experiments were conducted at least three times independently. The experimental data were statistically analyzed using an unpaired two‐tailed *t* test. *P* < 0.05 was considered statistically significant.

## Results

### *Helq* ablation impairs the induction of mPGCLCs

**Fig. 1:**
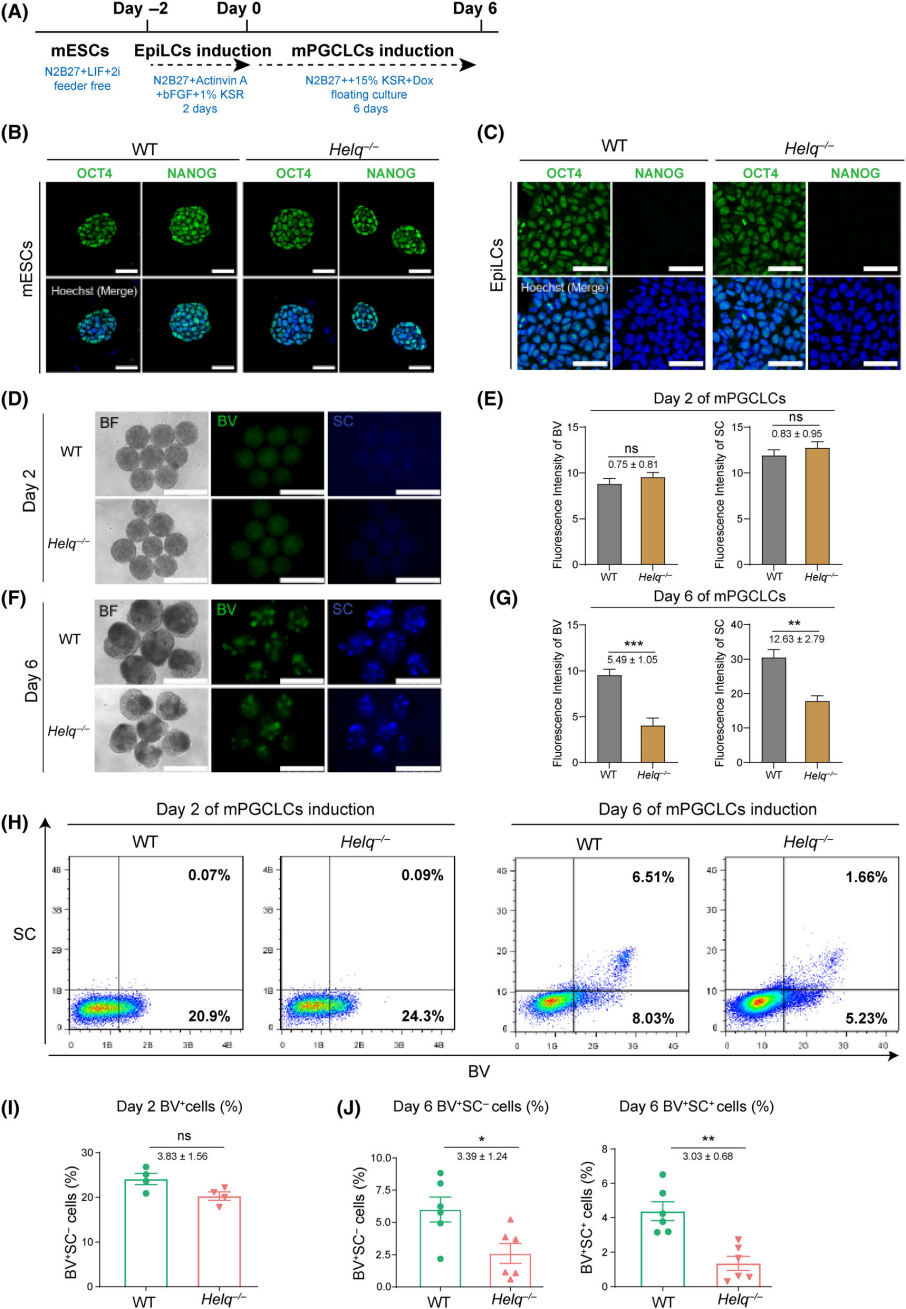
*Helq* ablation impairs the induction of mPGCLCs. (A) Scheme for the induction of mPGCLCs from mESCs. (B) Immunostaining for pluripotency markers OCT4 and NANOG in WT and *Helq* ^−/−^ mESCs. The nuclei were stained with Hoechst. Scale bars = 50 μm. (C) Immunostaining for OCT4 and NANOG in WT and *Helq* ^−/−^ EpiLCs. The nuclei were stained with Hoechst. Scale bars = 50 μm. (D, E) Expression and statistical analysis of BV in WT and *Helq* ^−/−^ EBs on days 2 of mPGCLCs. Scale bars = 500 μm; *n* = 8 (i.e. the number of EBs). ns, not significant; unpaired two‐tailed *t* test. (F, G) Expression and statistical analysis of *Blimp1*‐mVenus and *Stella*‐ECFP reporters (BVSC) in WT and *Helq* ^−/−^ EBs on day 6 of mPGCLCs. Scale bars = 500 μm; *n* = 6, mean ± SEM. **P < 0.01, ***P < 0.001; unpaired two‐tailed *t* test. (H) FACS analysis of the BVSC expression in WT and *Helq* ^−/−^ EBs at day 2 (left) and day 6 (right). (I) The proportion of BV^+^ cells in WT and *Helq* ^−/−^ EBs at day 2. The results were performed with four biologically independent experiments; *n* = 8 in each independent experiment, mean ± SEM. ns, not significant; unpaired two‐tailed *t* test. (J) The proportion of BV^+^SC^−^ (left) and BV^+^SC^+^ (right) cells in WT and *Helq* ^−/−^ EBs at day 6. The results were performed with six biologically independent experiments; *n* = 8 in each independent experiment, mean ± SEM. *P < 0.05, **P < 0.01; unpaired two‐tailed *t* test.

To explore the role of *Helq* in the generation of mPGCs, we derived *Helq* ^−/−^ mESCs as described previously [33], which harbored the *Blimp1*‐mVenus and *Stella*‐ECFP reporters (referred to as BV and SC, respectively) and contained Dox‐inducible transgenes of three transcription factors, *Blimp1*, *Prdm14* and *Tfap2c*. The *Blimp1*‐mVenus and *Stella*‐ECFP reporters strain could faithfully recapitulate endogenous *Prdm1* and *Dppa3* expression, enabling the tracing of germ cell generation upon specification [38]. Using this system, EpiLCs could be efficiently induced into mPGCLCs after the addition of Dox [20, 34] (Fig. 1A). In this process, the specified mPGCLCs could be first identified as BV‐positive (BV^+^) and further differentiated into the established mPGCLCs as BV‐ and SC‐positive cells (BV^+^SC^+^).

First, we did not detect off‐target effect from *Helq* knockout mESCs (Fig. S1). Subsequently, we examined the impact of *Helq* deficiency on the pluripotency of mESCs. Immunofluorescence analysis showed that no significant difference in the expression of *OCT4* and NANOG could be detected between *Helq* ^−/−^ and WT *groups*, indicating that pluripotency was unaffected in *Helq* ^−/−^ mESCs (Fig. 1B). Next, we aimed to determine whether *Helq* ^−/−^ would affect the ability of pluripotent cells into the germline. To this end, we induced mESCs into EpiLCs using culture conditions adapted from a previously reported protocol [20, 34]. Both WT and *Helq* ^−/−^ mESCs were primed to EpiLCs as shown by a decrease in the pluripotency marker at day 2, suggesting that the absence of *Helq* did not affect the induction into EpiLCs (Fig. 1C).

Subsequently, we proceeded with the floating culture of EpiLCs in an N2B27‐based differentiation medium containing 15% KSR and Dox. At day 2, a subset of cells began expressing BV and the fluorescence intensity was comparable in the WT and *Helq*‐null EBs (Fig. 1D,E). By day 6, a population of cells displayed BV^+^SC^+^ in both the WT and *Helq*‐null EBs. Notably, the fluorescence intensity of BV and SC was weaker in the *Helq*‐null group compared to the WT group (Fig. 1F,G).

Consistently, FACS analyses revealed that the entire EBs transitioned towards a weakly positive state for BV on day 2 (Fig. 1H). There was no significant difference between WT (24.1%) and *Helq*‐null (20.3%) groups, indicating that *Helq* deficiency did not impact early mPGCLCs specialization (Fig. 1I). By day 6, a subset of cells was BV^+^SC^+^. Strikingly, the proportion of BV^+^SC^−^ and BV^+^SC^+^ populations was significantly lower in the *Helq*‐null group (BV^+^SC^−^: 2.6%; BV^+^SC^+^: 1.4%) compared to the WT group (BV^+^SC^−^: 6.0%; BV^+^SC^+^: 4.4%) (Fig. 1H‐J). These results suggest that *Helq* loss‐of‐function can impair the induction of mPGCLCs.

### Repairing the mutation at single allele in the *Helq* ^−/−^ line can significantly restore the efficiency of mPGCLCs generation

**Fig. 2:**
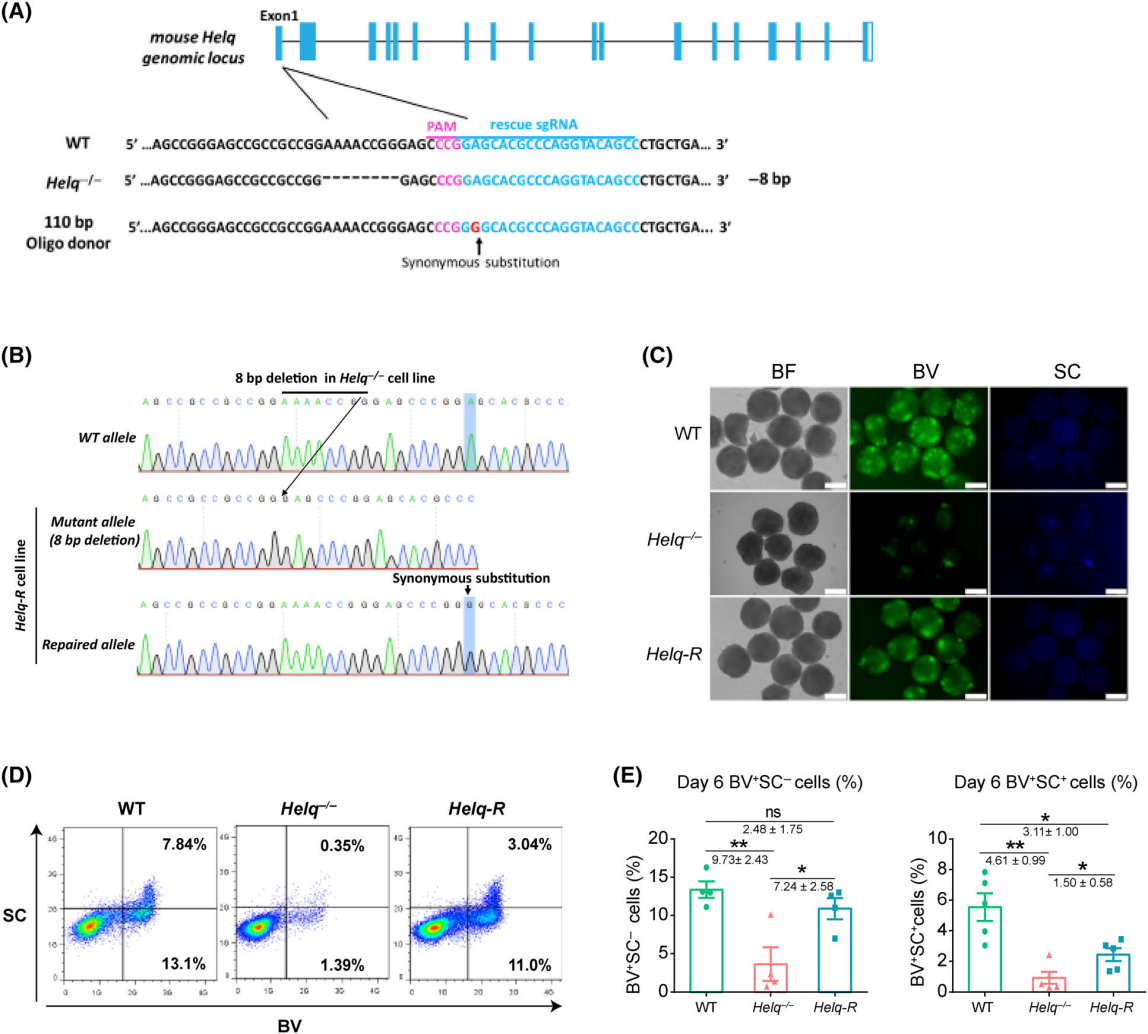
Repairing mutation at single allele in *Helq* ^−/−^ line can substantially restore the efficiency of mPGCLCs generation. (A) Scheme for correction of the mutation of *Helq* gene in *Helq* ^−/−^ mESCs using the CRISPR Cas9 system. The guide RNA sequence is marked by a blue line, and the protospacer‐adjacent motif (PAM) sequence is labeled in rose red. (B) Sequences of the alleles in WT and *Helq‐*R mESCs. *Helq‐*R mESCs carry a repaired allele and a mutation allele. (C) Expression of *Blimp1*‐mVenus and *Stella*‐ECFP reporters (BVSC) in WT, *Helq* ^−/−^ and *Helq*‐R EBs on day 6 of the induction of mPGCLCs. Sclale bars = 100 μm. (D) FACS analysis of the BVSC expression in WT, *Helq* ^−/−^ and *Helq*‐R EBs on days 6 of the induction of mPGCLCs. (E) Statistical analysis for the proportion of BV^+^SC^−^ (left) and BV^+^SC^+^ (right) cells in WT, *Helq* ^−/−^ and *Helq*‐R EBs at day 6. The results were performed with five biologically independent experiments; *n* = 8 in each independent experiment, mean ± SEM. *P < 0.05, **P < 0.01, ns, not significant; unpaired two‐tailed *t* test.

To further confirm the role of *Helq* in the induction of mPGCLCs, we next aimed to correct the *Helq* mutation in *Helq*‐null mESCs using CRISPR/Cas9‐mediated genome editing [37]. We designed a sgRNA and a single‐stranded DNA oligo as a template for homologous recombination‐based gene repair (Fig. 2A). The DNA sequencing results showed that the mutation has been corrected at one allele in the repaired mESCs line (*Helq‐*R) (Fig. 2B). As expected, a significant increase in the efficiency of BV^+^SC^−^ and BV^+^SC^+^ populations was observed in the *Helq‐*R mESCs derived EBs (BV^+^SC^−^: 10.9%; BV^+^SC^+^: 2.4%) compared to the *Helq* ^−/−^ mESCs derived EBs (BV^+^SC^−^: 3.7%; BV^+^SC^+^: 0.9%) (Fig. 2C‐E). Collectively, repairing the mutant sites at single allele in the *Helq* ^−/−^ line can significantly restore the efficiency of mPGCLCs generation.

### Loss of *Helq* leads to higher proportion of cell apoptosis at day 6 of induction of mPGCLCs

**Fig. 3:**
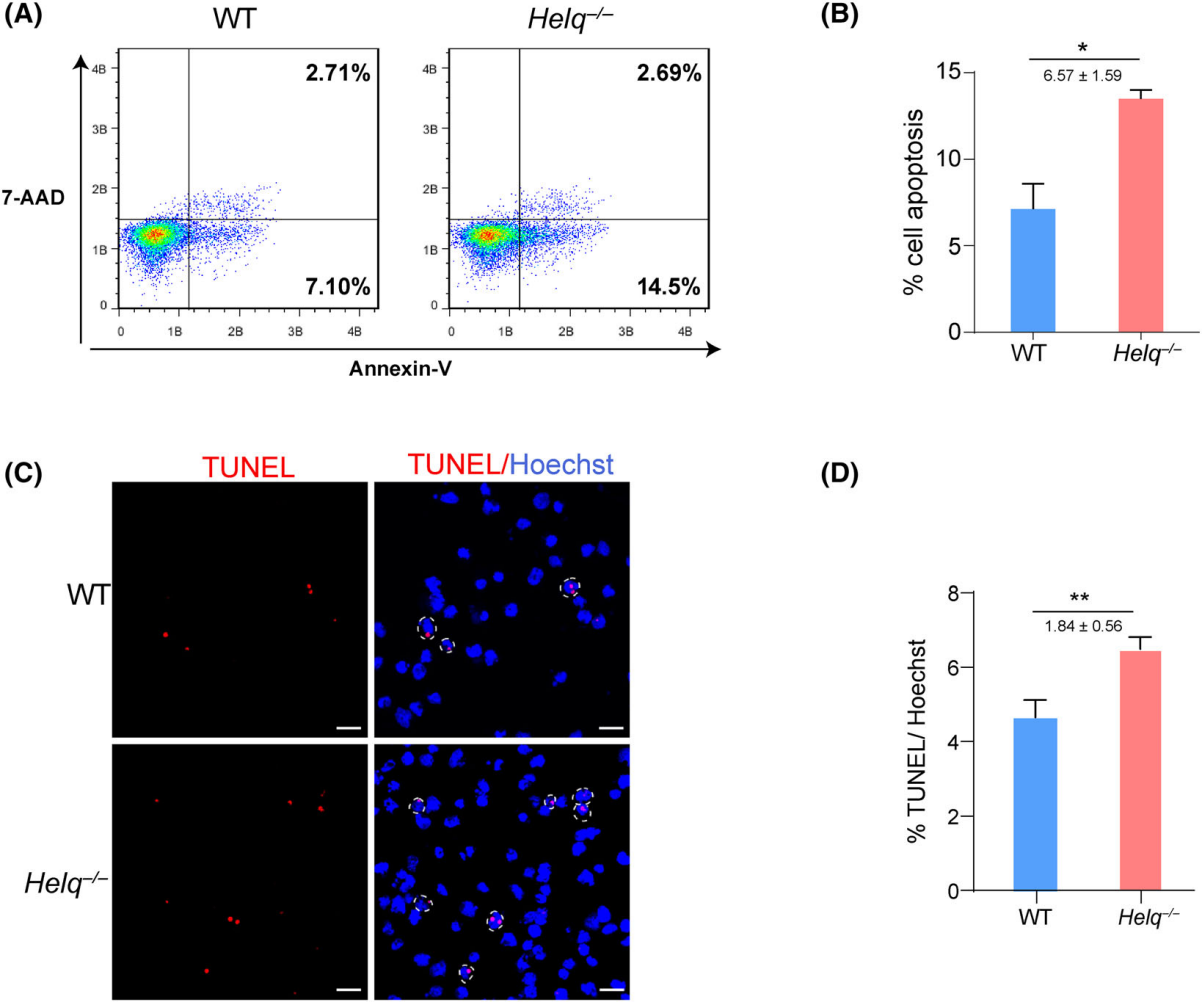
Loss of *Helq* leads to higher proportion of cell apoptosis at day 6 of induction of mPGCLCs. (A) Cell apoptosis was examined by Annexin V/7‐AAD FACS analysis in WT and *Helq* ^−/−^ EBs at day 6 of the induction of mPGCLCs. (B) Statistical analysis for the percentage of apoptotic cells in WT and *Helq* ^−/−^ EBs at day 6 of the induction of mPGCLCs. The results were performed with three biologically independent experiments; *n* = 8 in each independent experiment, mean ± SEM, *P < 0.05; unpaired two‐tailed *t* test. (C) TUNEL staining was performed in WT and *Helq* ^−/−^ EBs at day 6 of the induction of mPGCLCs. Scale bars = 20 μm. (D) Statistical analysis for the percentage of TUNEL positive cells in WT and *Helq* ^−/−^ EBs at day 6 of the induction of mPGCLCs; *n* = 4 in each independent experiment, nine fields of view from three independent experiments, mean ± SEM. **P < 0.001; unpaired two‐tailed *t* test.

Given the critical role of *Helq* in the DNA repair, the accumulation of DNA damage is likely to occur in *Helq* deficient cells. Additionally, it is well‐established that the germline maintains a stringent genomic integrity checkpoint, which would trigger apoptosis to eliminate aberrant cells [4, 39, 40]. Thus, we considered whether the loss of *Helq* would lead to apoptosis. To this end, we used the Annexin V‐FITC/7‐AAD apoptosis detection kit to examine the cell apoptosis event during WT and *Helq*‐null induction of mPGCLCs. On average, at day 6 with respect to induction of mPGCLCs, about 13.6% Annexin V^+^ 7‐AAD^−^ cells were detected in *Helq*‐null group, whereas the percentage of apoptotic cells in the WT group was < 7.0%, which reflected a significant increase in apoptotic cells in *Helq*‐null EBs (Fig. 3A,B). Statistical analysis of TUNEL staining also showed that the apoptosis rate in the *Helq*‐null group was significantly higher than that of the WT group (Fig. 3C,D). Consequently, *Helq* ^−/−^ can ultimately result in cell apoptosis during induction of mPGCLCs.

### p53 inhibitor can partially rescue the efficiency of the induction of mPGCLCs from the *Helq* ^−/−^ line

**Fig. 4:**
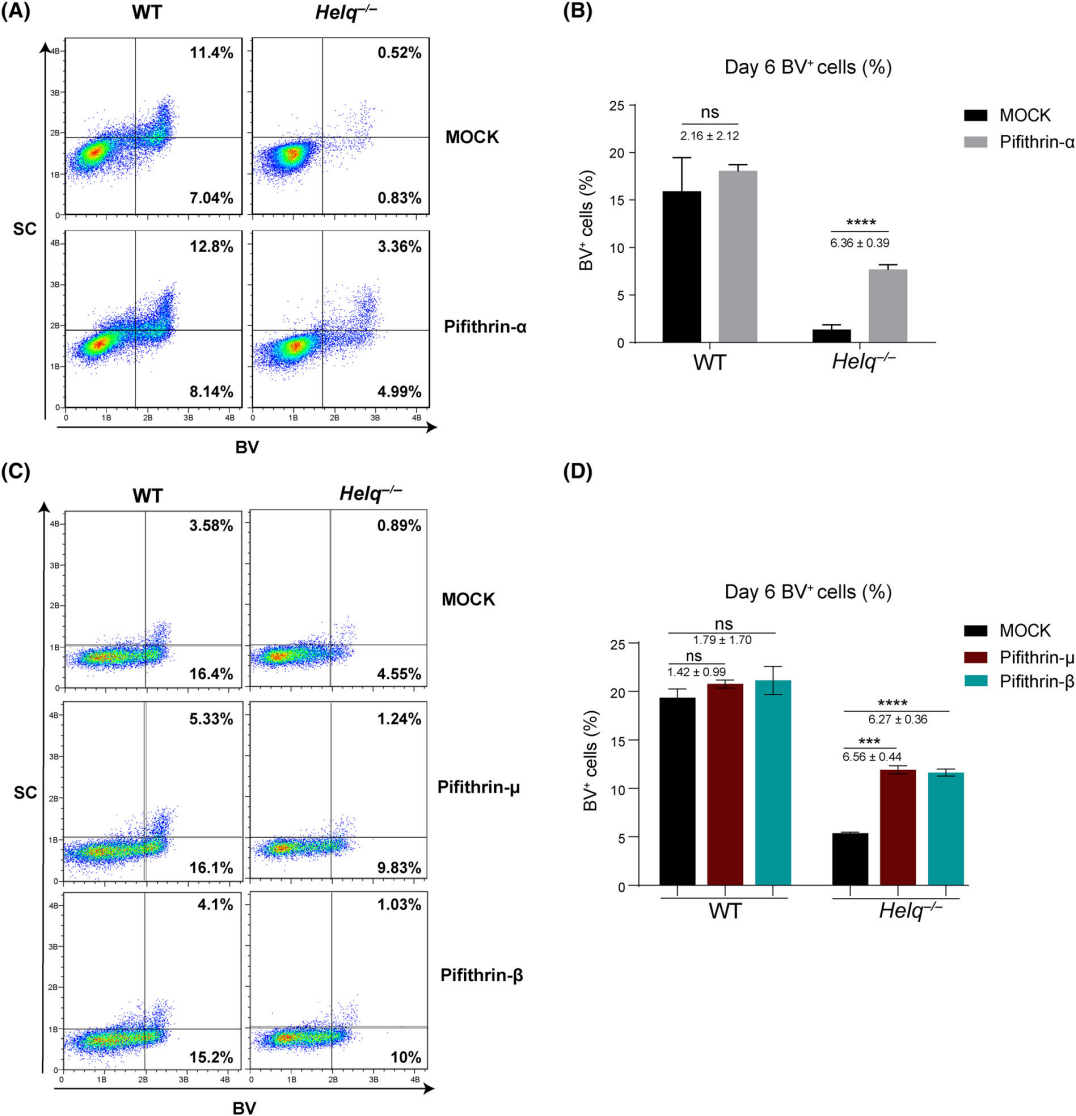
p53 inhibitor can partially rescue the efficiency of the induction of mPGCLCs in the *Helq* ^−/−^ line. (A) FACS analysis of the *Blimp1*‐mVenus and *Stella*‐ECFP reporters (BVSC) expression in WT and *Helq* ^−/−^ EBs at day 6 with DMSO (Mock) or pifithrin‐α treatment upon the induction of mPGCLCs. (B) Statistical analysis for the percentage of BV^+^ cells in WT and *Helq* ^−/−^ EBs at day 6 with Mock or pifithrin‐α treatment upon the induction of mPGCLCs. The results were performed with three biologically independent experiments; *n* = 8 in each independent experiment, mean ± SEM; ns, not significant, ****P < 0.0001; unpaired two‐tailed *t* test. (C) FACS analysis of the BVSC expression in WT and *Helq* ^−/−^ EBs at day 6 with Mock or pifithrin‐μ and pifithrin‐β treatment upon the induction of mPGCLCs. (D) Statistical analysis for the percentage of BV^+^ cells in WT and *Helq* ^−/−^ EBs at day 6 with Mock or pifithrin‐μ and pifithrin‐β treatment upon the induction of mPGCLCs. The results were performed with three independent experiments; *n* = 8 in each independent experiment, mean ± SEM; ns, not significant, ***P < 0.001, ****P < 0.0001; unpaired two‐tailed *t* test.

The key tumor suppressor p53 was the first determinant of susceptibility to DNA damage‐induced apoptosis to be identified [41]. The p53 signaling pathway is a well‐known, crucial and most common modulator of cell apoptosis. Thus, to define whether p53 signaling pathway was involved in *Helq*‐null induced cell apoptosis in mPGCLCs, we utilized the small molecule inhibitor, pifithrin‐α, to inhibit p53 signaling during the induction of mPGCLCs. We found that treatment with pifithrin‐α had no significant effect on the proportion of BV^+^ (including BV^+^SC^−^ and BV^+^SC^+^) cells in WT EBs at day 6 (Fig. 4A,B). However, in *Helq*‐null EBs with pifithrin‐α treatment, the proportion of BV^+^ cells increased from 1.35% to 8.35% (Fig. 4A,B). Moreover, to ensure the specificity of the p53 inhibitor, we selected two analogues of pifithrin‐α: pifithrin‐μ and pifithrin‐β. Consistently, the treatment with either pifithrin‐μ or pifithrin‐β led to a significant increase in the induction of mPGCLCs from the *Helq* ^−/−^ line. (Fig. 4C,D). Based on these data, p53 inhibition can partially rescue the efficiency of the induction of mPGCLCs from the *Helq* ^−/−^ line.

### The absence of *HELQ* significantly decreases the efficiency of the induction of hPGCLCs

**Fig. 5:**
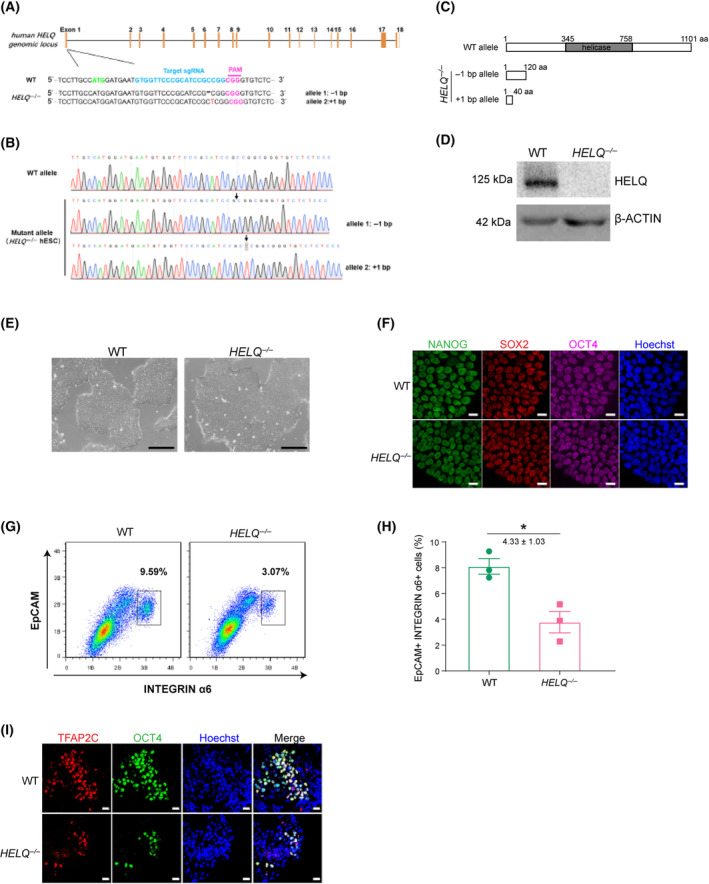
The absence of *HELQ* significantly decreases the efficiency of human PGCLCs (hPGCLCs) induction. (A) Schematic of the Cas9/sgRNA‐targeting site in the human *HELQ* genomic locus. The guide RNA sequence is marked by a blue line, and the PAM sequence is labeled in rose red. (B) Sequences of the alleles in WT and *HELQ* ^−/−^ human embryonic stem cells (hESCs). (C) The HELQ protein is truncated in *HELQ* ^−/−^ hESCs. (D) *HELQ* ^−/−^ is confirmed in hESCs by western blot analysis. (E) Bright‐field images of WT and *HELQ* ^−/−^ hESCs. Scale bars = 250 μm. (F) Co‐staining of pluripotency markers OCT4, SOX2 and NANOG showed no difference in expression in both WT and *HELQ* ^−/−^ hESCs. The nuclei were stained with Hoechst. Scale bars = 20 μm. (G) FACS analysis of the percentage of EpCAM^+^INTEGRINa6^+^ cells at day 4 EBs induced from hESCs through iMeLCs in WT and *HELQ* ^−/−^. (H) The proportion of EpCAM^+^INTEGRINa6^+^ cells in day 4 WT and *HELQ* ^−/−^ EBs. The results were performed with three biologically independent experiments; *n* = 8 in each independent experiment, mean ± SEM; *P < 0.05; unpaired two‐tailed *t* test. (I) Immunofluorescence of TFAP2C and OCT4 in WT and *HELQ* ^−/−^ EBs at induction of day 4. The nuclei were stained with Hoechst. Scale bars = 20 μm.

To examine the impact of *HELQ* deficiency on the induction of hPGCLCs, we generated the *HELQ* ^−/−^ hESCs line using the CRISPR‐Cas9 technique. One allele with 1‐bp deletion and the other allele with 1‐bp insertion in the first exon led to the frameshift mutation of the coding sequence of the *HELQ* gene, which resulted in the production of the truncated HELQ protein (Fig. 5A–C). Western blot analysis confirmed the absence of *HELQ* in knockout hESCs (Fig. 5D). The *HELQ* ^−/−^ hESCs displayed a characteristic morphology and expressed pluripotency marker genes (Fig. 5E,F). Subsequently, we induced the differentiation of hESCs into hPGCLCs using a previously established protocol. EpCAM and INTEGRINa6 have been identified as surface markers for hPGCLCs purification *in vitro* [21]. FACS analyses showed that the proportion of INTEGRINa6^+^/EpCAM^+^ cells was significantly decreased in the *HELQ* ^−/−^ hPGCLCs (3.8%) compared to the WT hPGCLCs (8.1%) (Fig. 5G,H). Further immunofluorescence analyses corroborated these findings by revealing a reduced percentage of cells positive for OCT4 and TFAP2C in *HELQ* ^−/−^ EBs (Fig. 5I). Together, these data suggest that the absence of *HELQ* affects the induction of hPGCLCs.

## Discussion

Up to 15% of couples are suffering from infertility and it has been estimated that almost 50% of infertility cases are a result of genetic defects [1, 42]. PGCs are the precursors of the germline and form the foundational population of germ cells that ensure reproductive capacity, heredity and evolutionary processes. However, investigating the molecular mechanisms underlying germ cell development is challenging because of limited access to early embryos, especially in human. *In vitro* systems, such as the induction system of mPGCLCs, and, more recently, hPGCLCs from human PSCs, have provided a platform and robust experimental materials for studying the physiology and pathology of germ cell development [12, 22, 23, 24, 35, 43].

Previous studies have demonstrated that disruption of the DNA damage repair mechanisms promote genomic instability, and lead to increased risks for cancer susceptibility and germline defects across species, such as fly, zebrafish, mouse and human [44, 45, 46, 47]. For example, the FA pathway is a dominant mechanism for inter‐strand cross‐link repair in vertebrates. Previous studies showed that FA patients and FA mutant mice were infertile or only rarely fertile [48, 49, 50]. *BLM* gene encodes a 3′‐5′ DNA helicase, which is required for precise double‐stranded DNA break repair, processing of DNA replication intermediates and rDNA metabolism. Impaired function of BLM caused shortened lifespan and reduced fertility in zebrafish, mouse and human [46, 51]. HELQ is also a crucial DNA helicase for maintaining genomic stability, which acts through a mechanism distinct from the function of FA pathway [29, 52]. A recent study investigating candidate variants in infertile men with Sertoli cell‐only syndrome shed light on the potential correlation between *HELQ* mutations and infertility [32]. However, whether HELQ is involved in PGCs specification and whether its roles in PGCs development are conserved between mouse and human remain unknown. In the present study, we revealed that the ablation of *Helq* significantly reduced the efficiency of mPGCLCs at day 6 of induction, whereas no apparent defects were observed in the mESCs and EpiLCs. This finding was consistent with our previous *in vivo* study, in which *Helq* ^−/−^ mESCs successfully contributed to diploid chimeric mice and subsequent self‐crossing of these chimeric mice resulted in the generation [33].

By utilizing the *in vitro* germ cell differentiation system, the present study demonstrated the impact of *Helq* on the efficiency of mPGCLCs generation at day 6 of induction. The mPGCLCs at this stage closely resemble *in vivo* E12.5 PGCs under the modified medium culture [34]. Consistently, our *in vitro* findings exhibit a remarkable concurrence with the previous observations from *in vivo* experiments, in which *Helq* ^−/−^ male mice displayed a reduced number of PGCs from E12.5 onwards [33], further supporting the importance of *Helq* in PGCs development. In addition, we discovered that repairing mutant sites at a single allele in *Helq* ^−/−^ line can significantly restore the efficiency of mPGCLCs generation. The efficiency was not a complete rescue in the *Helq*‐R line, in line with previous observations noting the haploinsufficiency of *Helq* [26]. These results suggest that our *in vitro* assays effectively simulate the impaired development of germ cells observed *in vivo* upon ablation of *Helq*.

Germline apoptosis, which occurs at multiple stages throughout PGCs development, is the crucial function ensuring the integrity of the germline [4, 40]. Previous studies have suggested the role of HELQ in the rescue of stalled forks in the S phase. They found increased persistent stalled forks and DNA damage in *HELQ*‐deficient cells [26, 29]. Therefore, we further examined whether the loss of *Helq* would lead towards an apoptotic fate. As expected, we observed increased apoptosis in *Helq*‐deficient embryonic bodies during the induction of mPGCLCs. Moreover, previous studies have revealed that HELQ exert functions in DNA repair and cell cycle checkpoint signaling by interacting with the RAD51 paralogs, XRCC2 and ATR [26]. Usually, in response to DNA damage, ATR is activated and phosphorylates the substrate CHK1 (p‐CHK1). Then p‐CHK1 further activates p53 to induce the cell apoptosis and inactivate inhibiting cyclin‐dependent kinases to arrest the cell cycle [41, 53, 54]. Consistently, we found that p53 inhibitor could partially rescue the efficiency of the induction of mPGCLCs from the *Helq* ^−/−^ line. In addition, a recent study reported the involvement of HELQ in ICL repair process based on the cellular co‐localization of ATR. They observed reduced p‐CHK1 in *HELQ* ^−/−^ U2OS cells treated with mitomycin C, and the accumulation of G2/M cells is also reduced [27]. By contrast, our previous study found a remarkable increase in p‐CHK1 positive germ cells in *Helq* ^−/−^ mice at E14.5 and there were more cells in the S phase of cell cycle in E11.5–E15.5 *Helq* ^−/−^ mitotic or transitional PGCs [33]. These results indicate that the mechanism of HELQ function might be different between somatic cells and germ cells.

Additionally, we generated *HELQ*‐deficient human ESC lines using CRISPR/Cas9 technology, and demonstrated that the absence of *HELQ* could also significantly decrease the efficiency of hPGCLCs induced from hESCs. Consistent with the influence of *Helq* on the process of the induction of mPGCLCs, no obvious difference was observed in the morphology and pluripotency of WT and *HELQ* ^−/−^ hESCs. These results indicate that genetic ablation of HELQ affects the induction of PGCLCs in a similar manner between mouse and human. Future elucidation of the precise molecular mechanisms underlying the function of HELQ in PGCs development, as well as the interplay between HELQ and other key factors involved in germ cell development, will deepen our understanding of infertility and potentially lead to improved diagnosis and treatment options for individuals facing reproductive difficulties.

In the present study, we reveal the conserved function of HELQ in PGCLCs induction between mouse and human, providing a fundamental resource for further investigations into the molecular mechanisms underlying germline differentiation and genetics studies on human fertility. Our study highlights the potential of utilizing *in vitro* models reconstituting mammalian germ cell development with respect to investigating the function of genes in reproductive biology.

## Conclusions

Our study sheds light on the involvement of HELQ in the induction of mouse and human PGCLCs. We revealed that the ablation of *Helq* significantly reduced the efficiency of mPGCLCs at day 6 of *in vitro* induction from mESCs. However, we did not observe apparent defects in the mESCs and EpiLCs upon *Helq* ablation. During the induction of mPGCLCs, we observed increased apoptosis in *Helq*‐deficient embryonic bodies. Notably, p53 inhibitor could partially rescue the efficiency of the induction of mPGCLCs from the *Helq* ^−/−^ line. Furthermore, to the best of our knowledge, our study represents the first evidence that the absence of *HELQ* significantly impacts the induction of hPGCLCs from hESCs, suggesting a potential conserved role for HELQ in PGCLCs induction between mouse and human. Future unraveling of the precise molecular mechanisms underlying HELQ's function in PGCs development, as well as the interplay between HELQ and other key factors involved in germ cell development, will deepen our understanding of infertility and potentially lead to improved diagnosis and treatment options for individuals facing reproductive difficulties.

## Conflicts of interest

The authors declare that they have no conflicts of interest.

## Author contributions

CW, YH, MW, CG, X‐YZ and Z‐ZT designed the study. CW, YH and XX performed the experiments and analyzed the data. CW, YH, FL, X‐YZ and Z‐ZT wrote the manuscript. All authors provided approval of the final version of the manuscript submitted for publication.

## Supporting information


**Fig. S1.** Identify the potential off‐target sites of *helq*.

## Data Availability

The data that support the findings of this study are contained within the article or Supporting information.
