# Correction to “HELQ deficiency impairs the induction of primordial germ cell‐like cells”

**DOI:** 10.1002/2211-5463.70029

**Published:** 2025-04-02

## Abstract

Cong Wan, Yaping Huang, Xingguo Xue, Gang Chang, Mei Wang, Xiao‐Yang Zhao, Fang Luo, Zhi‐Zhong Tang. HELQ deficiency impairs the induction of primordial germ cell‐like cells. *FEBS Open Bio*. 2024; 14(7): 1087–1100, https://doi.org/10.1002/2211‐5463.13810

The original designation of first authorship was incorrect: Cong Wan was listed as the sole first author, while Yaping Huang was inadvertently omitted as a co‐first author.

The authorship should have been amended to: Co‐first authors: Cong Wan & Yaping Huang.

We apologize for this error.

Cong Wan, Yaping Huang, Xingguo Xue, Gang Chang, Mei Wang, Xiao‐Yang Zhao, Fang Luo, Zhi‐Zhong Tang. HELQ deficiency impairs the induction of primordial germ cell‐like cells. *FEBS Open Bio*. 2024; 14(7): 1087–1100, https://doi.org/10.1002/2211‐5463.13810

The original designation of first authorship was incorrect: Cong Wan was listed as the sole first author, while Yaping Huang was inadvertently omitted as a co‐first author.

The authorship should have been amended to: Co‐first authors: Cong Wan & Yaping Huang.

We apologize for this error.

