# Supplementary material for: HELQ deficiency impairs the induction of primordial germ cell‐like cells
Source: FEBS Open Bio. 2024 May 8;14(7):1087–100. doi: 10.1002/2211-5463.13810 (PMC11216937; doi:10.1002/2211-5463.13810)
Supplement: Supplementary file 1 — Fig. S1. Identify the potential off‐target sites of helq. [file FEB4-14-1087-s001.pdf]

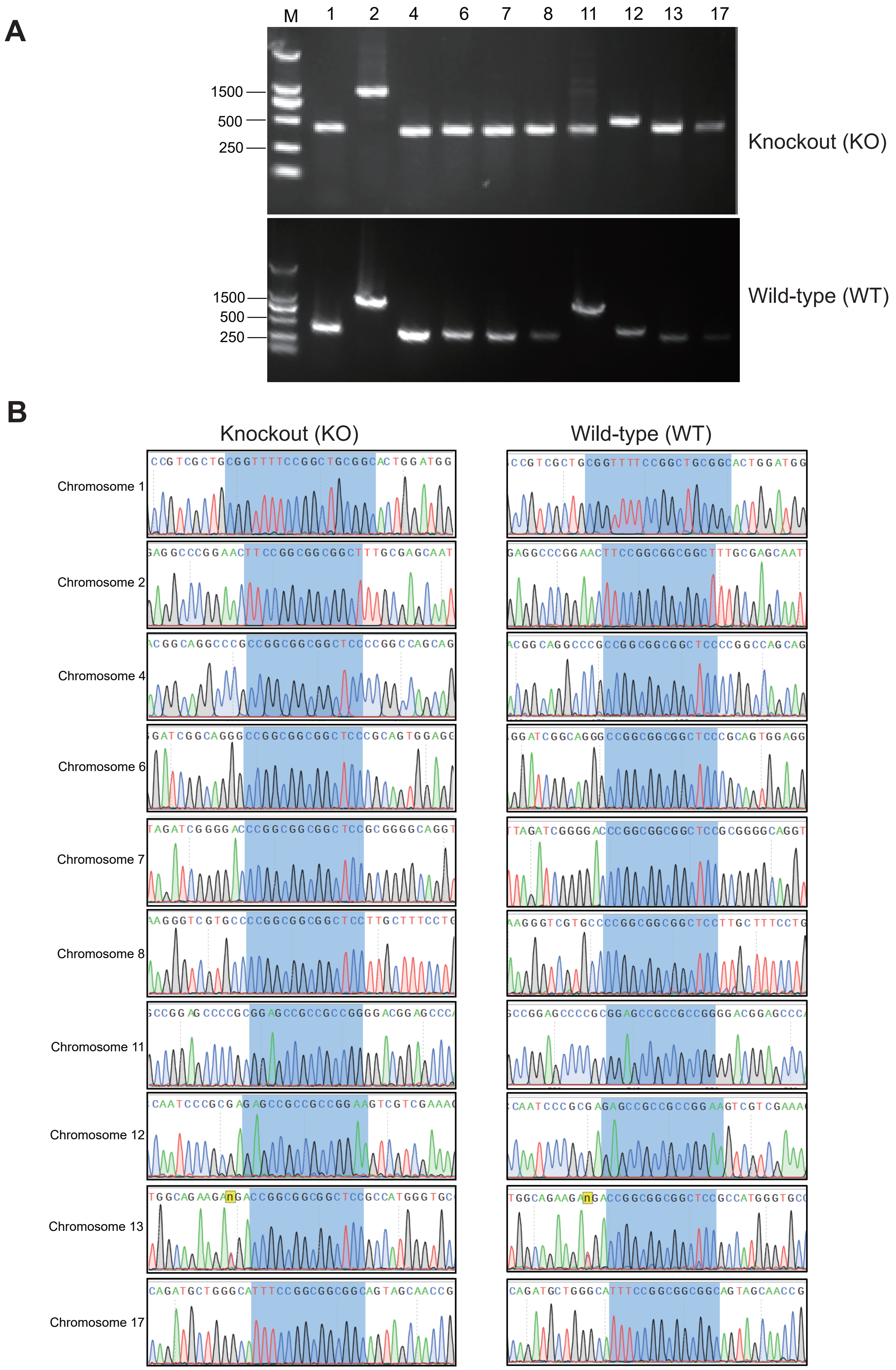

Figure S1. Identify the potential off-target sites of *helq* A) Agarose gel electropherograms of PCR amplification products. B) The sequencing chromatogram for the predicted off-target regions amplified from wild-type (WT) and knockout (KO). Blue regions indicate DNA fragments that are homologous to gRNA.
